# Adapting federated radiomics models for radiation pneumonitis prediction in patients receiving thoracic radiotherapy with immunotherapy

**DOI:** 10.3389/fimmu.2026.1793039

**Published:** 2026-04-07

**Authors:** Zirui Zhu, Meng Yan, Wenhao Ji, Zhen Zhang, Andre Dekker, Leonard Wee, Tian Zhang, Xiaojing Lai

**Affiliations:** 1 Zhejiang Key Laboratory of Particle Radiotherapy Equipment Department of Radiation Oncology, Zhejiang Cancer Hospital, Hangzhou Institute of Medicine (HIM), Chinese Academy of Sciences, Hangzhou, China; 2 Postgraduate Training Base Alliance of Wenzhou Medical University, Zhejiang Cancer Hospital, Hangzhou, China; 3 Department of Radiation Oncology, Key Laboratory of Cancer Prevention and Therapy, Tianjin Medical University Cancer Institute & Hospital, National Clinical Research Center for Cancer, Tianjin’s Clinical Research Center for Cancer, Tianjin, China; 4 Department of Radiation Oncology (Maastro), GROW Research Institute for Oncology and Reproduction, Maastricht University Medical Centre+, Maastricht, Netherlands

**Keywords:** federated learning, immunotherapy, radiation pneumonitis, radiomics, transfer learning

## Abstract

**Background and purpose:**

Radiation pneumonitis (RP) is one of the major dose-limiting toxicities of thoracic radiotherapy. Although multiple studies have attempted to predict RP, robust multicenter model development is often hindered by privacy regulations and data-transfer constraints, and many existing models are primarily derived from radiotherapy-alone populations, limiting applicability to contemporary regimens that incorporate immunotherapy. Therefore, this study aimed to develop an RP prediction model within a federated learning framework, incorporating sequential transfer learning strategies to enable separate risk assessment for radiotherapy patients with and without immunotherapy.

**Methods:**

Multicenter cohorts of lung cancer patients treated with definitive thoracic radiotherapy with or without immunotherapy were retrospectively collected and stratified by immunotherapy exposure. Radiomics features were extracted from whole-lung regions on pretreatment planning CT scans to construct RP prediction models. A federated learning framework was first applied to non-immunotherapy patients to learn common features of radiation pneumonitis without sharing raw data. The pretrained federated model was then sequentially transferred to immunotherapy treatment cohorts, with targeted fine-tuning to adapt to treatment specific RP patterns. Model performance was evaluated through internal validation and independent external validation, with SHAP analysis exploring feature importance differences across treatment settings.

**Results:**

A total of 610 patients were included from five multicenter cohorts. Using patients without immunotherapy for model development, the federated baseline model showed stable discrimination in external validation across non-immunotherapy cohorts (AUC = 0.77). When this baseline model was directly applied to the immunotherapy cohort without adaptation, performance dropped markedly (AUC = 0.43). After fine-tuning on immunotherapy data, the immunotherapy-adapted model achieved improved performance within the immunotherapy cohort (AUC = 0.76) and remained robust in an independent external immunotherapy validation cohort (AUC = 0.75). Feature attribution analysis showed a shift in model coefficients between immunotherapy-treated and non-immunotherapy patients.

**Conclusion:**

A federated modeling framework with treatment adaptation improves RP risk prediction across heterogeneous treatment settings under multicenter data constraints, particularly in immunotherapy-treated patients.

## Introduction

Lung cancer is among the most common malignancies worldwide and remains a leading cause of cancer-related mortality (1). The combination of radiotherapy and immunotherapy has emerged as a pivotal clinical strategies (2, 3). However, this therapeutic combination is associated with a increased risk of radiation pneumonitis (RP). Prior studies have reported an RP incidence of approximately 20%–25% following radiotherapy alone, which rises substantially when radiotherapy is combined with immunotherapy (4–6). In the PACIFIC trial, sequential immunotherapy after radiotherapy was associated with a higher incidence of pneumonitis compared with radiotherapy alone (7). These findings highlight the urgent need for predictive tools that enable early and individualized RP risk assessment in patients receiving combined therapy.

Recent advances in machine learning and deep learning have shown promise for RP prediction (8, 9). By extracting quantitative features from pretreatment CT images, radiomics based models can capture lung tissue heterogeneity and have shown improved predictive performance in several single-center studies (10, 11). Nevertheless, current radiomics- and deep learning–based RP prediction models remains limited. Most models are developed in non-immunotherapy settings and assume relatively homogeneous treatment backgrounds, which may not hold in patients receiving combined radiotherapy and immunotherapy. In addition, these models are often trained on single-center or small cohorts, and their generalizability and reproducibility are constrained by variations in imaging acquisition, reconstruction, and segmentation.

Due to data privacy and regulatory limitations, centralized sharing of raw imaging and radiotherapy data across institutions is often impractical, further hindering robust multicenter model development (12). Federated learning (FL) has emerged as an effective solution to address data privacy and multicenter collaboration challenges by enabling local data retention while leveraging multicenter information for model training (13). In an FL framework, models are trained locally at each institution, and only model parameters are shared and aggregated into a global model, allowing multicenter information to be used without direct data exchange (14).However, Fl alone does not account for heterogeneity introduced by different treatment backgrounds.

Therefore, in this study, we constructed a FL framework that further integrated treatment adaptation to enable RP risk prediction in both patients treated with immunotherapy or without immunotherapy. By integrating radiomics based imaging representations within this framework, our method aims to improve RP risk assessment across heterogeneous treatment settings in multicenter, real-world data environments.

## Methods

### Study design and datasets

**Figure 1:**
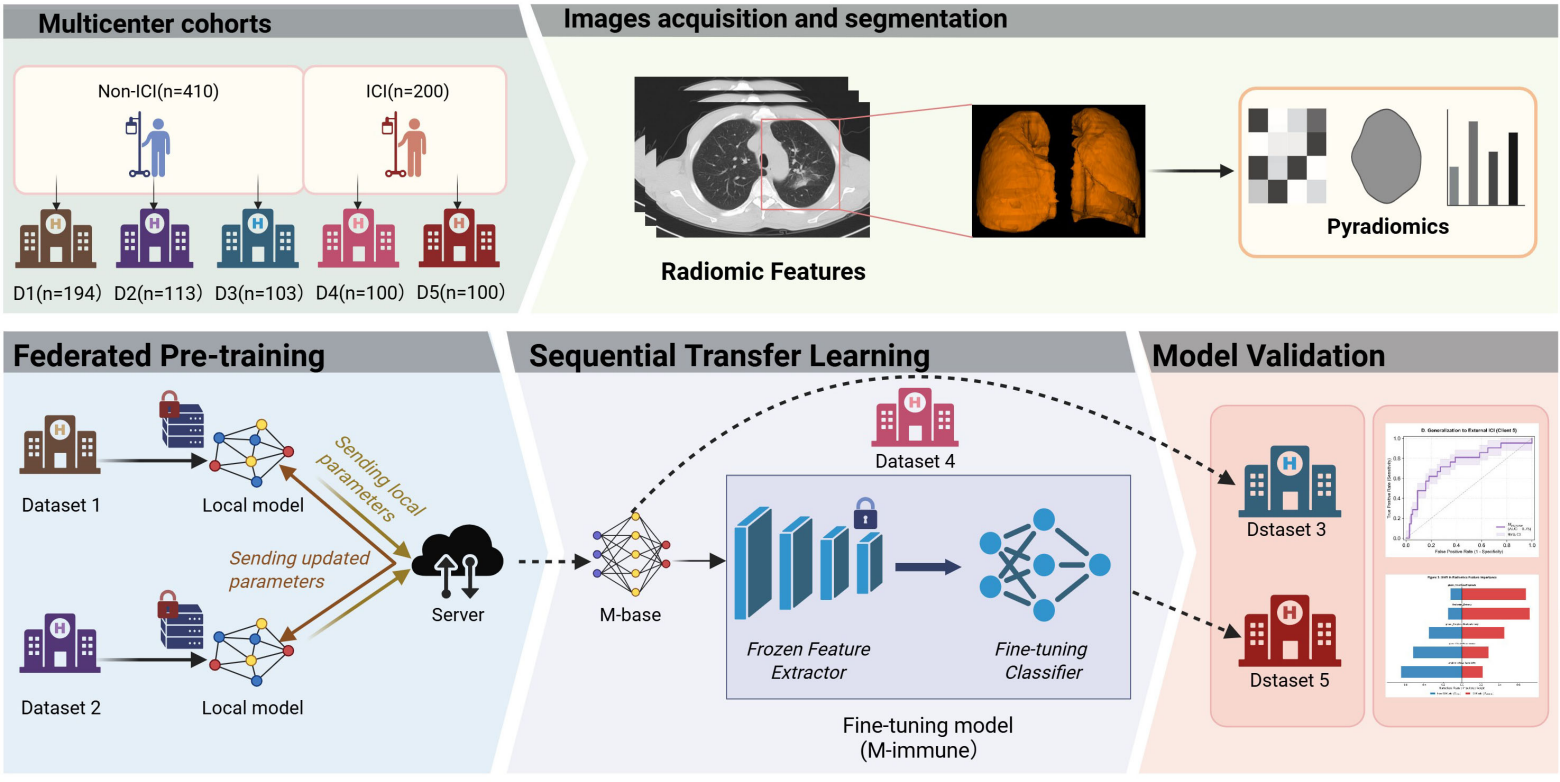
Study design. Multicenter cohorts were collected and divided into non-ICI and ICI populations. Chest CT images were acquired, lung regions were segmented, and radiomic features were extracted using PyRadiomics. A federated learning (FL) strategy was applied to pretrain a baseline model (M-base) on non-ICI cohorts (Dataset 1 and Dataset 2) without sharing raw data. The pretrained model was subsequently transferred to the ICI cohort (Dataset 4) via sequential transfer learning with a frozen feature extractor and fine-tuned classifier, resulting in an immune-specific model (M-immune). Model performance was evaluated on independent datasets (Dataset 3 and Dataset 5). ICI, immune checkpoint inhibitor; Non-ICI, patients who did not receive immune checkpoint inhibitor therapy; D1–D5, Dataset 1 to Dataset 5; CT, computed tomography; FL, federated learning; M-base, baseline model pretrained on non-ICI cohorts; M-immune, fine-tuned model for ICI-treated patients.

This study was approved by the ethics committee of Tianjin Medical University Cancer Institute and Hospital (IRB: bc20240049) and Zhejiang Cancer Hospital (IRB-2025-1756). The overall study workflow is illustrated in **Figure 1**. To protect data privacy, a federated learning framework was adopted to conduct cross-center model training without sharing raw data. The model integrated radiomics features from radiotherapy planning CT scans and constructed prediction models applicable to both immunotherapy treated and untreated patients across different treatment settings.

To assess the risk of radiation pneumonitis (RP) in patients with and without immunotherapy, we assembled five datasets. Dataset 1–3 comprised patients treated with definitive radiotherapy without immunotherapy and were used for model training, internal validation, and external testing, respectively. Dataset 4 and 5 included patients treated with combined thoracic radiotherapy and immunotherapy and served as the immunotherapy dataset training and external testing datasets, respectively. Dataset 1 was derived from the RTOG 0617 clinical trial, whereas Dataset 2, Dataset 3 and Dataset 4 were collected from Tianjin Medical University Cancer Institute and Hospital. Dataset 5 were obtained from Zhejiang Cancer Hospital. Detailed inclusion and exclusion criteria of Dataset1–5 and dataset definitions are provided in **Supplementary Materials 1**.

### Endpoint

The primary endpoint was defined as grade ≥2 RP according to the Common Terminology Criteria for Adverse Events (CTCAE) v4.0 (15). In Dataset 1, grades were abstracted from the trial case report forms. In Dataset 2-5, two experienced radiation oncologists assessed and graded RP occurrence based on follow-up CT scans, blood tests, and clinical symptom evaluation.

For patients undergoing thoracic radiotherapy, suspected pneumonitis events were first evaluated to exclude infectious etiologies based on microbiological testing, laboratory findings, and clinical response to anti-infective therapy. Non-infectious pneumonitis was then assessed according to radiation dose distribution, temporal relationship to radiotherapy and immunotherapy, clinical presentation, and imaging characteristics. Radiation pneumonitis (RP) was defined as pneumonitis occurring within 6 months after radiotherapy with imaging findings anatomically corresponding to the irradiated lung volume, particularly high-dose regions. Immune-related pneumonitis (IRP) was considered when inflammatory changes predominantly outside the radiation field or occurred outside the typical radiation-associated time window during ICI exposure. In cases with overlapping features, final classification was determined by multidisciplinary team (MDT) consensus. Only events adjudicated as RP were included as study endpoints.

### Image acquisition, lung segmentation and preprocessing

All patients planning CT scans were initially extracted in DICOM format. To ensure consistency across multicenter CT data, all images underwent standardized preprocessing, including resampling to 1×1×1mm³ and voxel intensities normalization to a range of −1000 to 400 HU. A 3D U-Net model was trained on Datasets 1 and 2 (N = 307) to automatically generate whole-lung masks, which were subsequently applied to the target datasets to delineate the regions of interest (ROIs) required for radiomics feature extraction. Scanner parameters are detailed in **Supplementary Material 2A**, and data preparation and preprocessing procedures are described in **Supplementary Material 2B**.

### Radiomic features extraction

Handcrafted radiomics features were subsequently extracted from these ROIs using the open-source PyRadiomics library (version 3.1.0). For each patient, 107 features were obtained, including first-order statistics, shape features, and multiple texture features (GLCM, GLRLM, GLSZM, NGTDM, and GLDM). The detailed list and categories of these radiomics features are summarized in **Supplementary Table 2**. Features were standardized using Z-score normalization based on the statistics of the training set.

### Prediction model

The predictive model used radiomics feature vectors as input and employed multiple fully connected layers to output the probability of radiation pneumonitis (RP) for each patient. Through nonlinear activation functions, the model learned complex relationships among features and ultimately performed binary classification to predict whether a patient would develop grade ≥2 RP. The network architecture and associated parameters are detailed in the **Supplementary Materials 3A**.

To assess the incremental value of radiomics features and to address potential confounding by inter-center heterogeneity in clinical practice, we predefined a conventional multivariable logistic regression model incorporating available clinical and dosimetric variables as a benchmark comparator. This clinical model was developed using the same training cohorts and evaluated on the identical internal and external test sets as the federated radiomics model, ensuring a fair and direct comparison. The performance of the clinical model was assessed with and without adjustment for Dataset to evaluate whether center-specific effects could account for differences in RP risk.

### Federated learning and sequential transfer learning

To enable multicenter model training without sharing raw imaging data, an FL approach was employed. During the federated pretraining phase, Datasets 1 and 2 independently trained models on local data without exchanging any patient images. In each FL round, clients only uploaded locally updated model parameters to a central server, which performed weighted aggregation based on client sample sizes to generate a global model (M-base). Federated pretraining was conducted for 50 global communication rounds, with five local training epochs performed at each participating center per round. Model optimization was performed using the Adam optimizer with an initial learning rate of 1×10^−3^ and a batch size of 16, and model parameters were aggregated using standard Federated Averaging weighted by client sample size. Convergence was monitored using validation AUC across communication rounds to ensure stable performance before completion of training. During sequential fine-tuning on the immunotherapy cohort, a reduced learning rate of 1×10^−4^ was adopted to facilitate stable adaptation. All federated experiments were implemented in a simulated cross-institution setting in which only model parameters were exchanged and no raw patient-level data were transmitted.

Given mechanistic differences in lung injury between patients receiving immunotherapy and those who do not, directly applying the baseline model to the immunotherapy cohort could degrade performance. Therefore, a sequential transfer learning strategy was implemented for model fine-tuning. The pre-trained M-base parameters were distributed to Dataset 4 and used as initial model parameters. Without transmitting any patient data, the feature extraction layers were frozen, and only the classification layers were fine-tuned, preserving general representations learned from non-immunotherapy patients while improving prediction performance for the immunotherapy cohort.

Given the high dimensionality of radiomic features and the modest sample size, particularly in the immunotherapy cohort, a relatively simple model architecture was adopted to mitigate overfitting risk. Sequential transfer learning constrained the hypothesis space by initializing model parameters from a larger source dataset, which improved convergence stability on the target cohort. Validation performance was monitored during training, and no divergence between training and validation performance was observed. Consistency between internal validation and independent external testing was examined to assess generalizability.

To address class imbalance between RP and non-RP cases, weighted sampling was applied during local training. Model discrimination was primarily evaluated using the area under the receiver operating characteristic curve, and precision-recall performance was additionally monitored during validation.

### Statistics

Model performance was evaluated using the area under the receiver operating characteristic curve (AUC), accuracy, sensitivity, and specificity. Calibration performance was additionally assessed in the test sets and is reported in the **Supplementary Materials**. 95% confidence intervals for each performance metric were estimated using 1,000 bootstrap resampling iterations. Model development and performance evaluation were conducted in Python 3.8.5 and R 4.0.5.

For patient baseline characteristics, continuous variables were presented as median (interquartile range) and compared between groups using the Kruskal–Wallis test. Categorical variables were expressed as counts (percentages) and compared using the Pearson χ² test, or the Fisher’s exact test when expected frequencies were less than 5. Clinical statistical analyses were performed using Statistical Package for Social Science program (SPSS for Windows, version 27.0; SPSS Inc, Chicago, IL).

## Results

### Patient characteristics

The study included 610 patients from five datasets. Patient characteristics are detailed in **Table 1**. Significant differences were observed among the datasets in terms of sex, age, pathological type, smoking history, treatment modality, and dosimetric parameters (p < 0.05), indicating inter-center heterogeneity.

**Table 1:** Patient characteristics across all datasets.

Characteristics	Overall N = 610	Dataset 1 N = 194	Dataset 2 N = 113	Dataset 3 N = 103	Dataset 4 N = 100	Dataset 5 N = 100	P-value
Gender							<0.001*
Female	161 (26.4%)	79.0 (40.7%)	26.0 (23.0%)	29 (28.2%)	16 (16.0%)	11 (11.0%)
Male	449 (73.6%)	115.0 (59.3%)	87.0 (77.0%)	74 (71.8%)	84 (84.0%)	89 (89.0%)
Age (years)							<0.001*
Median (IQR)	63.0 (56.0,69.0)	64.0 (56.0,69.0)	62.0 (53.0,69.0)	61.0 (54.0,66.0)	64.0 (58.0,69.0)	67.0 (59.0,72.0)
Pathology							<0.001*
SCC	245 (40.2%)	75 (38.7%)	53 (46.9%)	27 (26.2%)	39 (39.0%)	51 (51.0%)
non-SCC	229 (37.5%)	119 (61.3%)	35 (31.0%)	26 (25.2%)	34 (34.0%)	15 (15.0%)
SCLC	136 (22.3%)	0.0 (0.0%)	25 (22.1%)	50 (48.5%)	27 (27.0%)	34 (34.0%)
Smoking status							<0.001*
YES	449 (73.6%)	167 (86.1%)	85 (75.2%)	72 (69.9%)	56 (56.0%)	69 (69.0%)
NO	122 (20.0%)	14 (7.2%)	28 (24.8%)	31 (30.1%)	18 (18.0%)	31 (31.0%)
unknown	39 (6.4%)	13 (6.7%)	0.0 (0.0%)	0.0 (0.0%)	26 (26.0%)	0.0 (0.0%)
Immunotherapy type							<0.001*
Consolidation imm.	66 (10.8%)	0.0 (0.0%)	0.0 (0.0%)	0.0 (0.0%)	33 (33.0%)	33 (33.0%)
Induction + consolidation imm.	62 (10.2%)	0.0 (0.0%)	0.0 (0.0%)	0.0 (0.0%)	27 (27.0%)	35 (35.0%)
No imm	410 (67.2%)	194 (100%)	113 (100%)	103 (100%)	0.0 (0.0%)	0.0 (0.0%)
Induction imm.	72 (11.8%)	0.0 (0.0%)	0.0 (0.0%)	0.0 (0.0%)	40 (40.0%)	32 (32.0%)
Induction chemotherapy							<0.001*
YES	360 (59.0%)	0.0 (0.0%)	108 (95.6%)	98 (95.1%)	67 (67.0%)	87 (87.0%)
NO	250 (41.0%)	194 (100%)	5 (4.4%)	5 (4.9%)	33 (33.0%)	13 (13.0%)
Concurrent chemotherapy							<0.001*
YES	322 (52.8%)	194 (100%)	46 (40.7%)	25 (24.3%)	42 (42.0%)	15 (15.0%)
NO	288 (47.2%)	0.0 (0.0%)	67 (59.3%)	78 (75.7%)	58 (58.0%)	85 (85.0%)
Consolidation chemotherapy							<0.001*
YES	312 (51.1%)	173 (89.2%)	42 (37.2%)	60 (58.3%)	15 (15.0%)	22 (22.0%)
NO	298 (48.9%)	21 (10.8%)	71 (62.8%)	43 (41.7%)	85 (85.0%)	78 (78.0%)
PTV (cc)							<0.001*
Median (IQR)	395.9 (271.3,526.4)	460.0 (316.0,640.4)	416.4 (329.1,512.4)	412.6 (309.6,547.2)	355.0 (250.0,470.6)	228.3 (130.3,423.5)
Lung V5 (%)							<0.001*
Median (IQR)	48.8 (41.5,54.7)	57.7 (47.5,68.9)	49.9 (44.0,50.3)	49.9 (44.0,53.4)	46.9 (42.0,49.8)	41.4 (36.2,49.0)
Lung V20 (%)							<0.001*
Median (IQR)	24.7 (20.8,27.9)	29.0 (24.6,34.2)	25.0 (21.8,26.7)	25.9 (22.5,27.8)	23.0 (19.4,25.2)	19.6 (13.3,23.9)
Mean lung dose (Gy)							<0.001*
Median (IQR)	13.80 (11.6,16.0)	16.5 (14.1,19.3)	13.2 (12.1,14.2)	13.9 (12.3,14.9)	12.6 (11.2,14.0)	10.2 (7.1,12.1)
RP grade							0.016*
<2	480 (78.7%)	162 (83.5%)	79 (69.9%)	81 (78.6%)	85 (85.0%)	73 (73.0%)
≥2	130 (21.3%)	32 (16.5%)	34 (30.1%)	22 (21.4%)	15 (15.0%)	27 (27.0%)

IQR, Interquartile Range; SCC, lung squamous cell carcinoma; non-SCC, Non-small-cell lung cancer and not lung squamous cell carcinoma; SCLC, small cell lung cancer; 3D-CRT, 3dimensional comformal radiation therapy; IMRT, intensity-modulated radiotherapy; VMAT, volumetric modulated arc therapy; imm, immunotherapy; RP, radiation pneumonitis; PTV, planning tumor volume.

*p-value below 0.05 was considered statistically significant. The differences in characteristics were evaluated by Kruskal-Wallis test for continuous variables or exact Fisher test for categorical variables.

When patients were further stratified based on whether they received immunotherapy (**Supplementary Table 1**), notable differences in multiple clinical characteristics were evident between the immunotherapy and non-immunotherapy cohorts. The two groups differed significantly in pathological type, chemotherapy regimen, target volume, and lung dose parameters, suggesting that the receipt of immunotherapy markedly influences the distribution of baseline characteristics. Moreover, the incidence of grade≥2 RP also varied across datasets (p = 0.016).These findings indicated that immunotherapy had a significant impact on patients’ clinical characteristics and treatment selection, resulting in clearly distinct baseline profiles between immunotherapy and non-immunotherapy cohorts. Consequently, directly applying predictive models developed from a single patient population to another cohort in multicenter clinical data posed challenges, highlighting the importance of the present study.

### Model performance evaluation

**Figure 2:**
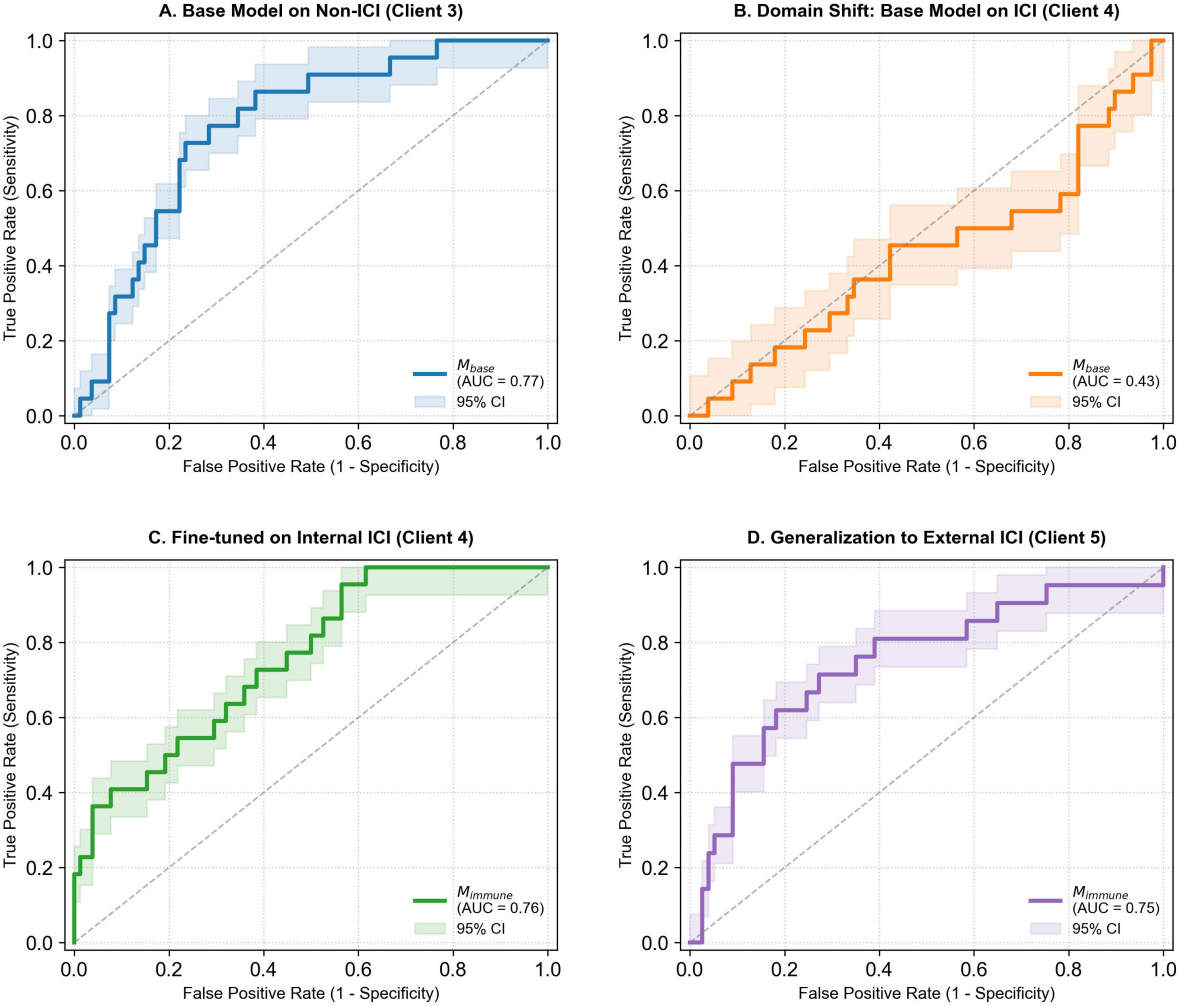
Receiver operating characteristic (ROC) curves across different datasets. Receiver operating characteristic (ROC) curves illustrating model performance for predicting radiation pneumonitis (RP) across different patient cohorts. **(A)** The baseline model (Mbase) performs well on non-ICI patients (Client 3) with an AUC of 0.77. **(B)** Applying the baseline model to ICI-treated patients (Client 4) causes a sharp performance drop (AUC = 0.43), highlighting the limitations of models trained on non-ICI cohorts. **(C)** Fine-tuning the baseline model on internal ICI data (Client 4) restores performance (Mimmune, AUC = 0.76), showing the benefit of domain adaptation. **(D)** The fine-tuned model generalizes well to an external ICI cohort (Client 5, AUC = 0.75), suggesting robust predictive ability across independent ICI-treated datasets. Shaded areas indicate 95% confidence intervals (CI). ROC, Receiver Operating Characteristic; AUC, Area Under the Curve; CI, Confidence Interval; RP, Radiation Pneumonitis; ICI, Immune Checkpoint Inhibitor; Non-ICI, patients who did not receive immune checkpoint inhibitor therapy; Mbase, Baseline model; Mimmune, Fine-tuned model for ICI patients; TPR, True Positive Rate; FPR, False Positive Rate; Client, Participating dataset in the federated learning framework.

Results of the receiver operating characteristic (ROC) curve are shown in **Figure 2**, with specific performance metrics listed in **Table 2**.

**Table 2:** Models’ performance across dataset 3 to 5.

Model	Test dataset	AUC (95% CI)	Accuracy	Sensitivity	Specificity
Mbase	Dataset 3 (Non-ICI)	0.77 (0.69-0.82)	0.72	0.71	0.73
Mbase	Dataset 4 (ICI)	0.43 (0.39-0.66)	0.55	0.42	0.61
Mimmune	Dataset 4 (ICI)	0.76 (0.73-0.88)	0.76	0.79	0.75
Mimmune	Dataset 5 (ICI)	0.75 (0.65-0.81)	0.69	0.67	0.7

Performance of the baseline and ICI-adapted models across independent test datasets.

Model performance was evaluated using AUC with 95% confidence intervals, accuracy, sensitivity, and specificity.

AUC, area under the receiver operating characteristic curve; CI, confidence interval; ICI, immune checkpoint inhibitor; Non-ICI, patients who did not receive immune checkpoint inhibitor therapy; Mbase, baseline model trained on No-ICI patients; Mimmune, ICI-adapted model trained on ICI-treatedpatients.

In patients not receiving immunotherapy, the baseline model (M-base) performed well in the external test cohort (Dataset 3), achieving an AUC of 0.77 (95% CI: 0.69–0.82) (**Figure 2A**). In this dataset, the model’s accuracy, sensitivity, and specificity were 0.72, 0.71, and 0.73, respectively.

However, when applied to patients receiving immunotherapy (Dataset 4), the baseline model showed reduced discrimination, with an AUC of 0.43 (95% CI: 0.39–0.66) and a sensitivity of 0.42 at the predefined threshold (**Figure 2B**).

The fine-tuned immune-specific model (M-immune) achieved significant performance improvement on the internal validation set for immunotherapy (Dataset 4). As shown in **Figure 2C**, the AUC of the immune-specific model on Dataset 4 increased to 0.76 (95% CI: 0.73–0.88), markedly outperforming the baseline model within the same dataset

To further validate the model’s robustness, external validation was conducted on the completely independent Dataset 5. The immune-specific model maintained stable predictive performance with an AUC of 0.75 (95% CI: 0.65–0.81) (**Figure 2D**) and a sensitivity of 0.67.

In contrast to the federated radiomics model, the clinical-dosimetric model demonstrated limited to no discriminative ability across all evaluation cohorts. Without center adjustment, the clinical model yielded AUCs ranging from 0.50 to 0.59 across the three external test sets (Dataset 3, 4, and 5). After including center ID as a covariate to account for inter-center heterogeneity, the model’s performance remained poor, with AUCs of 0.50, 0.53, and 0.52 for Dataset 3, 4, and 5, respectively. Detailed performance metrics and ROC curves for the clinical models are provided in **Supplementary Table 3** and **Supplementary Figure 2**.

### Feature contribution and importance analysis

**Figure 3:**
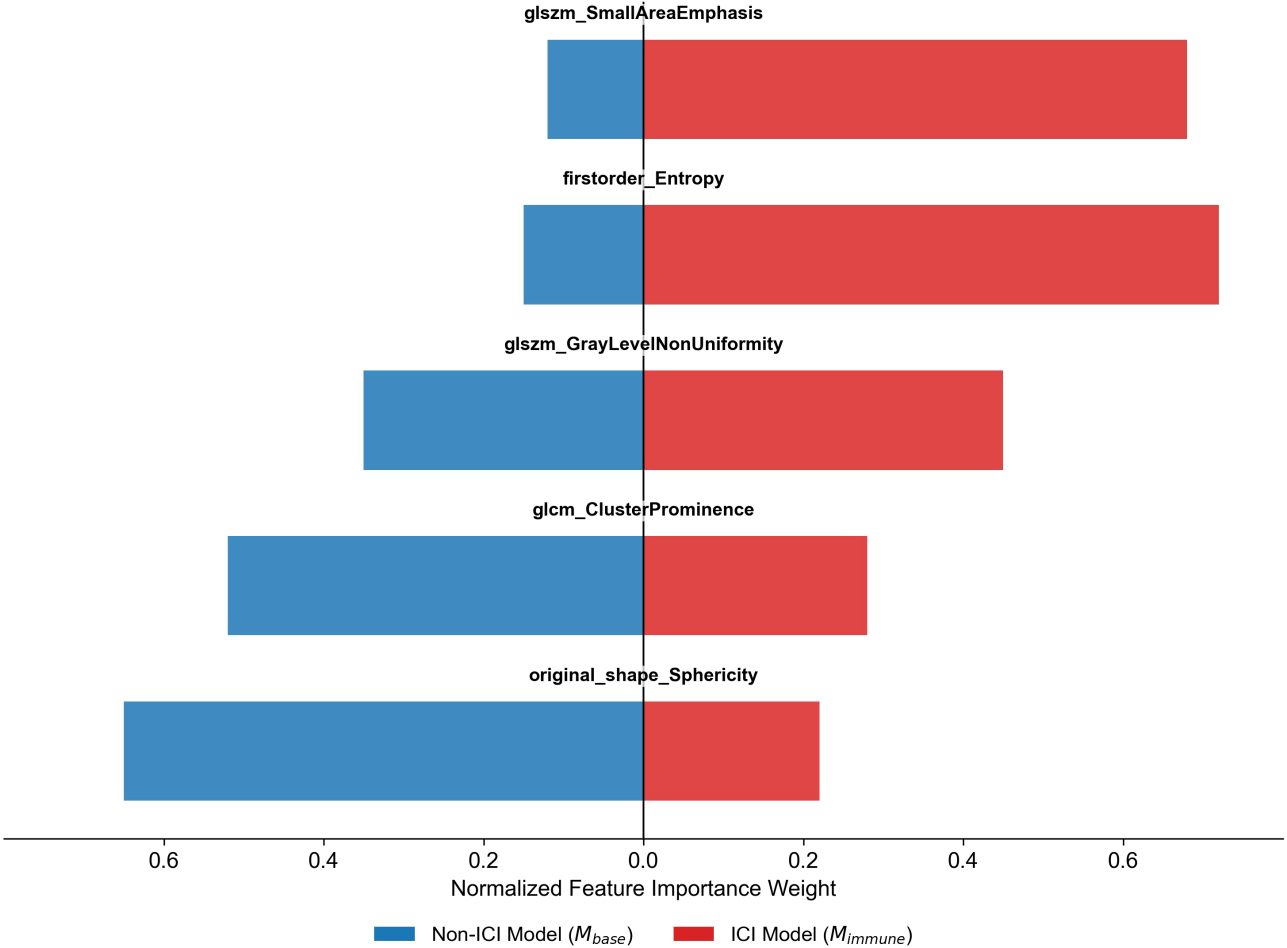
Radiomics features importance across Mbase and Mimmune. Comparison of normalized feature importance weights for selected radiomics features between the Non-ICI model (Mbase, blue bars) and the ICI model (Mimmune, red bars). Positive values indicate higher importance in the ICI model; negative values indicate higher importance in the Non-ICI model. Notably, feature relevance shifts when accounting for immune checkpoint inhibitor (ICI) treatment. Texture-related features, such as glszm_SmallAreaEmphasis and firstorder_Entropy, gain importance in the ICI model. In contrast, shape- and cluster-related features, like original_shape_Sphericity and glcm_ClusterProminence, dominate in the Non-ICI model. These results highlight treatment-specific alterations in radiomics signatures associated with radiation pneumonitis risk. ICI, Immune Checkpoint Inhibitor; Non-ICI, patients who did not receive immune checkpoint inhibitor therapy; Mbase, Baseline model; Mimmune, Fine-tuned model for ICI patients; GLCM, gray level co-occurrence matrix; GLSZM, gray level size zone matrix; GLCM and GLSZM are second-order texture matrices used to characterize intratumoral heterogeneity.

To further investigate the predictive mechanisms of the models, we ranked radiomics features using SHAP values (**Figure 3**). Compared to the non-immunotherapy model, the importance of radiomic features in the immunotherapy-specific model showed significant changes. In the non-immunotherapy model, morphological and structure-related features (such as sphericity and cluster prominence) contributed more to model predictions. In the immune-specific model, features reflecting textural heterogeneity (e.g., entropy and small region emphasis) exhibited markedly increased importance.

## Discussion

In this study, we developed a federated learning framework for RP risk prediction using multicenter patient data, enabling privacy-preserving without sharing raw data. By integrating radiomics features, the framework supports RP risk prediction in both patients receiving and not receiving immunotherapy through treatment model adaptation, enabling its applicability across different treatment settings.

Previous studies have attempted to predict RP using machine learning and deep learning methods, primarily based on single-center cohorts to develop radiomics, dosimetric, and deep learning feature models. For instance, a model integrating PET/CT, CT radiomics features, and deep learning features demonstrated high predictive performance in lung cancer patients (16); a deep learning model combining chest CT with clinical and dose features was developed for RP risk prediction (17); additionally, dose-based radiomics using 3D dose distributions was combined with deep learning-based radiomics features for prediction (18).Systematic reviews indicate that existing machine learning and deep learning approaches for RP prediction demonstrate generally good model performance, yet are often constrained by sample size limitations and insufficient generalization capabilities (12). Therefore, this study employs a federated learning framework to integrate real-world data from multiple centers while preserving privacy. This approach enhances model generalization, yields more robust predictions, and improves clinical applicability. In supplementary analyses, we observed that models relying solely on structured clinical and dosimetric variables exhibited limited discrimination (AUC < 0.6) across all test cohorts, regardless of center adjustment. This finding is notable for two reasons. First, it suggests that the inherent heterogeneity in clinical documentation and treatment practices across real-world centers may introduce substantial noise, limiting the generalizability of traditional clinical models. Second, and more critically, the superior and consistent performance of the federated radiomics framework (AUC > 0.70) indicates that imaging-derived features capture phenotypic information directly relevant to radiation pneumonitis that is not reflected in routine clinical or dosimetric parameters. While future integration of harmonized clinical variables may further enhance model interpretability, our results demonstrate that the federated learning of radiomics features alone can yield a robust, center-agnostic biomarker for predicting radiation pneumonitis in diverse clinical settings.

We will discuss patients who have received immunotherapy separately from those who have not, as multiple clinical studies and systematic reviews have demonstrated that patients receiving immunotherapy combined with radiotherapy exhibit a higher overall incidence of RP compared to those undergoing chest radical radiotherapy alone (19–22).In patients receiving chest radical radiotherapy alone, RP development is typically closely related to dose distribution, with inflammatory responses often confined to the irradiation field. In contrast, patients undergoing combined radiotherapy and immunotherapy may experience pulmonary injury across a broader, more diffuse distribution, leading to more complex clinical and radiographic presentations (23, 24). Mechanistically, immune checkpoint inhibitors and radiotherapy can both induce inflammatory responses in lung tissue through overlapping immune pathways, which may amplify radiation-related lung injury and extend inflammation beyond the irradiated field (25, 26). Consequently, the lung injury resulting from combination therapy may differ from RP caused by radiotherapy alone in terms of temporal sequence, spatial distribution, and severity. Such biological heterogeneity may attenuate the association between dose-correlated imaging features and RP risk, which may partly contribute to the reduced predictive performance observed in ICI-treated patients. In addition, immunotherapy-related pneumonitis (IRP) may present with atypical radiological patterns, such as cryptogenic organizing pneumonia-like manifestations, which overlap with but are not entirely identical to classical RP. These nuanced imaging presentations may increase the risk of misclassification when applying models trained predominantly on conventional RT-induced RP patterns. Moreover, jointly analyzing immunotherapy-treated and non-immunotherapy patients may obscure treatment-specific mechanisms of RP, potentially limiting the model’s ability to learn discriminative features relevant to different therapeutic contexts.

In the immunotherapy-naive population, the M-base model demonstrated robust performance in the independent external validation cohort (D3) after training and internal validation within the training cohorts (D1 and D2). This is expected, as the external validation cohort D3 shares considerable consistency with the training cohort D2 in terms of treatment regimens and racial composition. This also indicates the model’s stable capability to assess RP in the context of conventional radical thoracic radiotherapy. However, when directly applying M-base to the immunotherapy-treated cohort (D4), the model’s predictive performance significantly deteriorated. These results indicate that the baseline model exhibited stable predictive performance in patients not receiving immunotherapy but declined in the immunotherapy dataset, suggesting limited generalizability across treatment contexts. This decline likely stems from immunotherapy altering the biological processes of lung injury. This shift in injury patterns renders the imaging features learned by the model in non-immunotherapy populations inapplicable to immunotherapy patients, resulting in a substantial reduction in the predictive capability of traditional RP models for immunotherapy-naive individuals.

To address the issue of reduced predictive performance of M-base in immunotherapy-treated populations, we introduced a sequential transfer learning strategy within a federated learning framework to tailor the model. The model was first pre-trained via federated learning on a multi-center cohort of non-immunotherapy patients to fully capture common radiographic features of radiation pneumonitis. Building upon this foundation, the pre-trained model was then applied to the immunotherapy cohort. While keeping the feature extraction layer parameters unchanged, the classification decision layer underwent fine-tuning to mitigate overfitting risks on the small immunotherapy dataset. Following this immunotherapy-specific refinement, the model demonstrated improved predictive performance on an independent external validation cohort (D5) of immunotherapy patients.

This two-stage modeling approach, which combines federated pre-training with sequential transfer learning, constitutes a key distinction from traditional federated learning methods. Unlike conventional approaches that directly train a unified model across all nodes, our method distinguishes between the commonalities of radiation pneumonitis and the specificities of the immunotherapy context. This enables the model to leverage the advantages of multi-center data while mitigating the risk of aggregating parameters derived from heterogeneous treatment contexts. While ensuring patient data privacy, this two-stage modeling approach enhances model stability and generalizability within immunotherapy populations, better aligning with clinical modeling needs across diverse treatment contexts.

Subsequently, we conducted SHAP analysis on feature importance (**Figure 3**). Compared to the M-base model, the importance of radiomic features reflecting texture heterogeneity significantly increased in the immunotherapy-specific model, while morphological and geometric features showed relatively reduced predictive importance. This shift suggests that in patients not receiving immunotherapy, the radiographic presentation of RP may be more closely associated with local structural alterations, with the model primarily relying on features reflecting lung tissue morphology and geometry for risk prediction. In contrast, within the immunotherapy cohort, features reflecting pulmonary parenchymal texture complexity and spatial heterogeneity gained importance. This likely reflects the more diffuse, heterogeneous inflammatory processes associated with lung injury following immunotherapy combined with radiotherapy, differing from the focal lesions typically seen with radiotherapy alone (27, 28).

This shift in feature importance partially explains the variation in predictive performance across different treatment settings, underscoring the necessity for personalized model adjustments in immunotherapy populations. Notably, our model relied primarily on handcrafted radiomics features designed to capture texture heterogeneity. While effective in conventional RP prediction, such predefined features may not fully differentiate subtle biological and radiographic distinctions between RP and IRP, particularly in early stages of toxicity. From a clinical practice perspective, follow-up for patients receiving immunotherapy combined with radiotherapy should not only focus on local changes in high-dose irradiation areas but also prioritize early alterations in overall pulmonary parenchymal texture, such as diffuse density irregularities or increased texture complexity in non-irradiated fields (29). Incorporating such radiographic information into risk assessment facilitates the identification of patients at high risk for pneumonia before clinical symptoms emerge, aiding physicians in early diagnosis and treatment strategy optimization.

This study has several limitations. First, as a retrospective analysis, it incorporates a multicenter cohort and undergoes external validation but cannot entirely avoid case selection bias and follow-up variability. Second, while the federated learning framework partially mitigates the generalization issues of single-center models, heterogeneity across centers in imaging parameters, radiotherapy techniques, and immunotherapy regimens may still impact model performance. Additionally, among patients receiving radiotherapy combined with immunotherapy, radiation pneumonitis and immune checkpoint inhibitor-associated pneumonitis may present with overlapping clinical and radiographic features, reflecting a diagnostic challenge in routine clinical practice. Despite our comprehensive assessment based on follow-up imaging, clinical symptoms, and treatment time windows, some degree of diagnostic uncertainty cannot be completely excluded. This limitation is inherent to retrospective studies in this clinical context. Fourth, the relatively limited sample size in the immunotherapy cohort precludes more refined subgroup analyses regarding different types of immune drugs or administration sequences. Finally, this study primarily relied on CT radiomics s for modeling, excluding other functional imaging modalities, molecular biological indicators, or inflammation-related biomarkers. This limitation restricts deeper exploration of the mechanisms underlying RP development. Our model relied primarily on handcrafted radiomics features designed to capture texture heterogeneity. While effective in conventional RP prediction, such predefined features derived from static structural and textural information on pretreatment CT images may not fully capture systemic immune activation or dynamic inflammatory processes associated with combination therapy. This constraint may partly limit model adaptability across different therapeutic contexts and contribute to the observed performance decline in the immunotherapy cohort. Future prospective, multicenter studies incorporating richer multimodal data are needed to further validate the model’s stability and clinical applicability.

## Conclusion

In summary, this study established and validated a multicenter RP prediction framework based on federated learning and sequential transfer learning strategies. The model demonstrated stability in populations without prior immunotherapy and achieved robust predictive performance in immunotherapy patients after targeted fine-tuning. Feature importance analysis revealed variations in RP-associated imaging characteristics across different treatment contexts, partially explaining model performance differences. These findings underscore the necessity of considering diverse treatment contexts in risk prediction and demonstrate the potential value of federated learning strategies for toxicity prediction in clinical practice. This approach facilitates the identification of high-risk patients prior to clinical symptom onset, providing valuable guidance for timely treatment strategy adjustments.

## Data Availability

Publicly available datasets were analyzed in this study. This data can be found here: Dataset 1 is available at TCIA (http://doi.org/10.7937/TCIA.2018.jze75u7v) (30, 31). Remaining datasets (2-5) are available from the corresponding author upon reasonable request and institutional ethical approval.
