# Supplementary material for: Adapting federated radiomics models for radiation pneumonitis prediction in patients receiving thoracic radiotherapy with immunotherapy
Source: Front Immunol. 2026 Apr 7;17:1793039. doi: 10.3389/fimmu.2026.1793039 (PMC13095741; doi:10.3389/fimmu.2026.1793039)
Supplement: Supplementary file 1 [file DataSheet1.pdf]

# Supplementary Material

## 1. Patient Cohorts and Datasets

Five distinct patient datasets were included in this study. Three datasets comprised patients treated at Tianjin Medical University Cancer Institute and Hospital: two cohorts of patients receiving thoracic radiotherapy without immunotherapy, and one cohort receiving combined thoracic radiotherapy and immunotherapy. The remaining two datasets included patients from the publicly available RTOG-0617 trial and an external immunotherapy cohort from Zhejiang Cancer Hospital. The study was approved by the Institutional Review Board (IRBbc20240049).

### *A. Patient Selection and Criteria of Dataset 1 to5*

Dataset 1 included 194 patients from the RTOG-0617 trial who received 60 Gy thoracic radiotherapy without immunotherapy and was used for model training and internal validation.

Dataset 2 (RT Cohort): The retrospective cohort included 113 patients who underwent definitive radiotherapy (RT) for lung cancer between January 2024 and December 2024 from Tianjin Medical University Cancer Institute and Hospital.

Dataset 3 (RT Cohort): The retrospective cohort included 103 patients who underwent definitive radiotherapy (RT) for lung cancer between January 2025 and December 2025 from Tianjin Medical University Cancer Institute and Hospital.

Dataset 4 ((RT + Immunotherapy Cohort): The retrospective cohort included 100 patients who underwent definitive radiotherapy (RT) for lung cancer between January 2024 and December 2024 from Tianjin Medical University Cancer Institute and Hospital.

Dataset 5 ((RT + Immunotherapy Cohort): The retrospective cohort included 100 patients who underwent definitive radiotherapy (RT) for lung cancer between January 2025 and October 2025 from Zhejiang Cancer Hospital.

Inclusion criteria
(1) Histologically confirmed diagnosis of non-small cell lung cancer (NSCLC) or small cell lung cancer (SCLC). (2) Clinical stage I-III NSCLC or limited-stage SCLC, as defined by the American Joint Committee on Cancer (AJCC) Staging Manual, 8th edition. (3) Treated with curative-intent thoracic RT. (4) No prior history of thoracic surgery or RT.

Exclusion criteria
(1) Poor quality planning CT images. (2) Significant treatment deviations, such as an interruption of more than five consecutive days during the RT. (3) Presence of a significant pulmonary infection within six months post-RT, which could confound the diagnosis of radiation pneumonitis. (4) Follow-up time less than 6 months. For Dataset 1 specifically: History of receiving immunotherapy either before or within the 6 months following the completion of thoracic RT.

### *B. Details for re-use of RTOG-0617 dataset 1*

Inclusion criteria

(1) Patients received full course of radiotherapy. (2) The thickness of CT images ranges from 1.25mm to 3mm. (3) Field of view is 500mm diameter and each axial image dimension should be 512 x 512, such that the final reconstructed per pixel spatial resolution falls between 0.9mm and 1.3mm. (4) Either IV contrast or non-IV contrast CT images.

Exclusion criteria

(1) Patients diagnosed with (infectious) pneumonia rather than radiation pneumonitis. (2) No corresponding CT images were available. (3) Abnormal CT images with same pixel values (-1024) for the whole lung. (4) If there were multiple DICOM plan and dose files, it was not possible to determine which one was applied was actually approved for the first fraction of RT. (5) CT images that failed to include the entirety of both lungs.

## 2. Data Preprocessing and Automated Segmentation

To ensure data consistency across multi-center cohorts, particularly for the external validation set (Client 5) where manual contours were unavailable, we implemented a standardized preprocessing pipeline. First, all CT images were resampled to a uniform voxel spacing of 1 × 1 × 1 mm3. Pixel intensities were normalized to the range of [−1000, 400] Hounsfield Units (HU) to highlight lung tissue textures.

For ROI delineation, we employed a domain-adapted automated segmentation strategy. A 3D U-Net model was pre-trained using the fully annotated source domain datasets (Clients 1 and 2, N = 307) to segment the whole lung volume. This auxiliary model was then deployed to inference the target domain data, generating masks for radiomics extraction. Hand-crafted radiomics features were extracted using the open-source PyRadiomics library. A total of 107 features were extracted per patient, including first-order statistics, shape features, and texture features (GLCM, GLRLM, GLSZM, NGTDM, GLDM). Z-score normalization was applied to the features based on the statistics of the training set.

## 3. Federated Learning Architecture

To address the challenge of data silos and privacy concerns, we constructed a federated learning framework based on a Multi-Layer Perceptron (MLP) backbone.

### *A. Model Architecture*

Given an input radiomics feature vector x ∈ $R^d$ (where d = 107), the model $f(x;\ \theta)$ aims to predict the probability of radiation pneumonitis (RP). The network consists of three fully connected layers. The hidden layers utilize the Rectified Linear Unit (ReLU) activation function

to capture non-linear relationships, while the output layer employs a Sigmoid function for binary classification. The forward propagation can be formulated as:

$$\hat{y} = \sigma(W_2 \cdot ReLU(W_1 x + b_1) + b_2) \tag{1}$$

where $\theta = \{W_1, b_1, W_2, b_2\}$ represents the trainable parameters, and $\hat{y}$ denotes the predicted probability of RP.

### *B. Federated Pre-training (Source Domain)*

We adopted the standard Federated Averaging (FedAvg) algorithm to train the base model ($M_{base}$) on the non-ICI cohorts (Client 1 and Client 2). The objective is to minimize the global loss function $F(\theta)$ without sharing raw patient data:

$$\min_{\theta} F(\theta) = \sum_{k=1}^{k} \frac{n_k}{N} F_k(\theta) \tag{2}$$

where $K = 2$ is the number of participating clients, $n_k$ is the sample size of client $k$, and $N = \sum n_k$ is the total sample size. $F_k(\theta)$ is the local empirical loss defined by binary cross-entropy:

$$F_k(\theta) = -\frac{1}{n_k} \sum_{i=1}^{n_k} [y_i \log(\hat{y_i}) + (1 - y_i) \log(1 - \hat{y_i})] \tag{3}$$

In each communication round $t$, the server distributes the global model $\theta^t$ to clients. Clients perform local updates $\theta_k^{t+1} \leftarrow \theta^t - \eta \nabla F_k(\theta^t)$ via Stochastic Gradient Descent (SGD) and upload the updates to the server for aggregation:

$$\theta^{t+1} = \sum_{k=1}^{k} \frac{n_k}{N} \theta_k^{t+1} \tag{4}$$

## 4. Sequential Transfer Learning (Target Domain)

Direct application of $M_{base}$ to immunotherapy (ICI) patients often yields suboptimal performance due to domain shift (i.e., differences in biological mechanisms between chemoradiotherapy and ICI-induced pneumonitis). To adapt the model, we employed a sequential transfer learning strategy. The pre-trained parameters from $M_{base}$ were transferred to the target ICI node (Client 4) to initialize the model $M_{immune}$. We applied a layer-freezing technique to prevent overfitting on the small-sample ICI dataset. Specifically, the weights of the feature extraction layers $(W_1, b_1)$ were frozen to retain the general representations of radiation injury learned from the large-scale source domain. Only the parameters of the classification head $(W_2, b_2)$ were fine-tuned using the local ICI data:

$$\theta_{head}^{new} = \theta_{head}^{base} - \eta' \nabla_{\theta_{head}} \mathcal{L}_{ICI} \tag{5}$$

This strategy allows the model to recalibrate the decision boundary for the immune-related cohort while leveraging the robust feature encoding from the non-immune population.

**Table 1. Comparison between ICI and Non-ICI**

| Characteristic | Overall N = 610 | non-ICI N=410 | ICI N=200 | P-value |
|---|---|---|---|---|
| **Gender** | | | | <0.001* |
| Female | 161 (26.4%) | 134 (32.7%) | 27 (13.5%) | |
| Male | 449 (73.6%) | 276 (67.3%) | 173 (86.5%) | |
| **Age (years)** | | | | 0.002* |
| Median (IQR) | 63.0 (56.0,69.0) | 62.0 (55.0,68.0) | 65.0 (59.0,71.0) | |
| **Pathology** | | | | <0.001* |
| SCC | 245 (40.2%) | 155 (37.8%) | 90 (45.0%) | |
| non-SCC | 229 (37.5%) | 180 43.9%) | 49 (24.5%) | |
| SCLC | 136 (22.3%) | 75 (18.3%) | 61 (30.5%) | |
| **Smoking Status** | | | | <0.001* |
| YES | 449 (73.6%) | 324 (79.0%) | 125 (62.5%) | |
| NO | 122 (20.0%) | 73 (17.8%) | 49 (24.5%) | |
| unknown | 39 (6.4%) | 13 (3.2%) | 26 (13.0%) | |
| **Immunotherapy type** | | | | <0.001* |
| Consolidation imm. | 66 (10.8%) | 0 | 66 (33.0%) | |
| Induction + consolidation imm. | 62 (10.2%) | 0 | 62 (31.0%) | |
| No imm | 410 (67.2%) | 410 (100%) | 0 | |
| Induction imm. | 72 (11.8%) | 0 | 72 (36.0%) | |
| **Induction chemotherapy** | | | | <0.001* |
| YES | 360 (59.0%) | 206 (50.2%) | 154 (77.0%) | |
| NO | 250 (41.0%) | 204 (49.8%) | 46 (23.0%) | |
| **Concurrent chemotherapy** | | | | <0.001* |

| | | | | |
|---|---|---|---|---|
| YES | 322 (52.8%) | 265 (64.6%) | 57 (28.5%) | |
| NO | 288 (47.2%) | 145 (35.4%) | 143 (71.5%) | |
| **Consolidation chemotherapy** | | | | <0.001* |
| YES | 312 (51.1%) | 275 (67.1%) | 37 (18.5%) | |
| NO | 298 (48.9%) | 135 (32.9%) | 163 (81.5%) | |
| **PTV (cc)** | | | | <0.001* |
| Median (IQR) | 395.9 (271.3,526.4) | 427.0 (318.6,560.1) | 293.5 (180.6,423.5) | |
| **Lung V5 (%)** | | | | <0.001* |
| Median (IQR) | 48.8 (41.5,54.7) | 50.6 (44.1,59.0) | 44.6 (38.3,49.0) | |
| **Lung V20 (%)** | | | | <0.001* |
| Median (IQR) | 24.7 (20.8,27.9) | 26.1 (22.9,29.5) | 21.3 (16.5,24.3) | |
| **Mean Lung Dose (Gy)** | | | | <0.001* |
| Median (IQR) | 13.80 (11.6,16.0) | 14.4 (12.8,16.5) | 11.8 (9.4,14.1) | |
| **RP Grade** | | | | 0.896* |
| ＜2 | 480 (78.7%) | 322 (78.5%) | 158 (79.0%) | |
| ⩾2 | 130 (21.3%) | 88 (21.5%) | 42 (21.0%) | |

Abbreviations: IQR = Interquartile Range; SCC = lung squamous cell carcinoma; non-SCC = Non-small-cell lung cancer and not lung squamous cell carcinoma; SCLC = small cell lung cancer; 3D-CRT=3dimensional comformal radiation therapy; IMRT = intensity-modulated radiotherapy; VMAT = volumetric modulated arc therapy; imm = immunotherapy; RP = radiation pneumonitis; PTV = planning tumor volume.

* p-value below 0.05 was considered statistically significant. The differences in characteristics were evaluated by Kruskal-Wallis test for continuous variables or exact Fisher test for categorical variables

**Table 2. Summary of the selected radiomics features**

| **Features** | **Definition** |
|---|---|
| original_glszm_LargeAreaHighGrayLevelEmphasis | It quantifies the joint distribution of large size zones and high gray-level values within the region of interest. |
| original_glszm_SizeZoneNonUniformityNormalized | It measures the variability of zone sizes throughout the image. |
| original_ngtdm_Contrast | It quantifies the local intensity variation between a voxel and its surrounding neighbors, captures spatial heterogeneity and texture coarseness. |
| original_firstorder_90Percentile | The high percentile (90th) of the intensity distribution in low-frequency components. |
| original_ngtdm_Coarseness | The coarseness of an image's texture. |

**Figure 1. Structure of the Multilayer Perceptron Model**

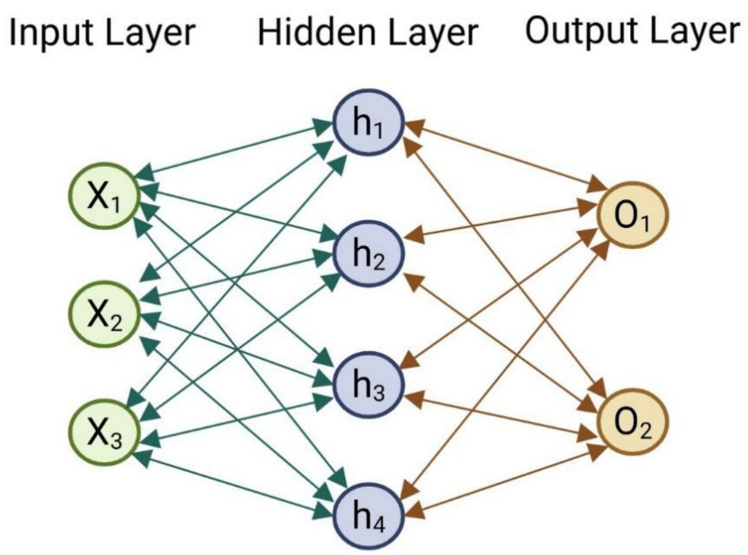


**Schematic illustration of a multilayer perceptron (MLP) network, consisting of an input layer, one hidden layer, and an output layer.**

**Table 3. Multivariable logistic regression analysis of clinical and dosimetric variables for radiation pneumonitis.**

| | Variable | OR (95% CI) | P value |
|---|---|---|---|
| **Without Dataset adjustment** | **Induction chemotherapy** | **0.83 (0.47 – 1.46)** | **0.527** |
| | **Mean lung dose** | **1.002 (1.001 – 1.004)** | **0.008** |
| | **Smoking status** | **1.42 (0.76 – 2.60)** | **0.261** |
| | **Consolidation chemotherapy** | **1.39 (0.81 – 2.38)** | **0.235** |
| **With Dataset adjustment** | **Induction chemotherapy** | **0.33 (0.07 – 1.07)** | **0.095** |
| | **Mean lung dose** | **1.003 (1.001 – 1.005)** | **0.002** |
| | **Smoking status** | **1.60 (0.83 – 3.00)** | **0.149** |
| | **Consolidation chemotherapy** | **1.87 (1.02 – 3.46)** | **0.044** |
| | **Dataset 2** | **0.40 (0.08 – 1.55)** | **0.217** |
| | **Dataset 3** | **0.26 (0.05 – 1.04)** | **0.077** |
| | **Dataset 4** | **0.22 (0.04 – 0.80)** | **0.035** |
| | **Dataset 5** | **0.13 (0.02 – 0.62)** | **0.017** |

Note: The table presents odds ratios (OR) with 95% confidence intervals (CI) forclinical and dosimetric predictors. Two models are shown: (1) without adjustment for center, and (2) with adjustment for center ID (using Dataset 1 as the reference).

**Figure 2.ROC curves of multivariable logistic regression models**

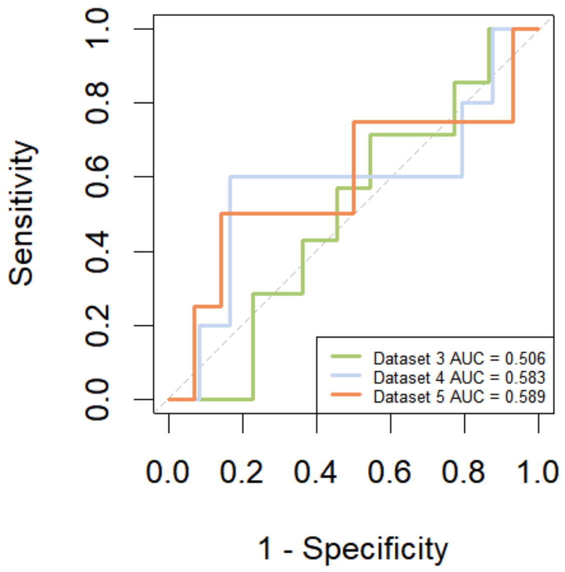


**(A)**

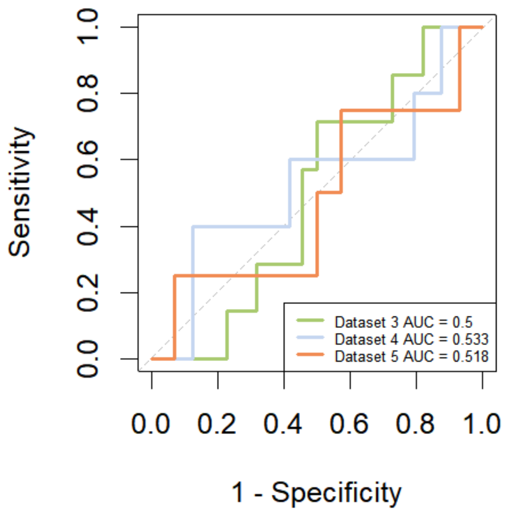


**(B)**

(A) ROC curves of the clinical model without center adjustment, stratified by center (Dataset 3, 4, 5) in the test set. The model demonstrated poor discriminative ability with AUCs of 0.506, 0.583, and 0.589 for Dataset 3, 4, and 5, respectively.

(B) ROC curves of the clinical model with center adjustment, stratified by center (Dataset 3, 4, 5) in the test set. After adjusting for center, the predictive performance remained poor, with AUCs of 0.5, 0.533, and 0.518 for dataparts 3, 4, and 5, respectively.

**Supplementary Table 4. Calibration performance across test sets.**

| Model | Dataset | Brier score |
|---|---|---|
| Mbase | Dataset 3 (Non-ICI) | 0.15 |
| Mbase | Dataset 4 (ICI) | 0.2 |
| Mimmune | Dataset 4 (ICI) | 0.15 |
| Mimmune | Dataset 5 (ICI) | 0.18 |

Brier scores of the baseline (Mbase) and immunotherapy-adapted (Mimmune) models across evaluation datasets. Lower Brier scores indicate better calibration.
